# “Limiting access to iron decreases infection of Atlantic salmon SHK-1 cells with bacterium *Piscirickettsia salmonis*”

**DOI:** 10.1186/s12917-021-02853-6

**Published:** 2021-04-13

**Authors:** Rodrigo Díaz, José Troncoso, Eva Jakob, Stanko Skugor

**Affiliations:** 1 Cargill Innovation Centre, Camino a Pargua km 57, Colaco km 5, Calbuco, Puerto Montt, Chile; 2 Cargill Innovation Centre, Dirdalsstranda 51, 4335 Dirdal, Norway

**Keywords:** Iron chelation, *Piscirickettsia salmonis*, SRS, Atlantic salmon, SHK-1, Gene expression

## Abstract

**Background:**

Vertebrate hosts limit the availability of iron to microbial pathogens in order to nutritionally starve the invaders. The impact of iron deficiency induced by the iron chelator deferoxamine mesylate (DFO) was investigated in Atlantic salmon SHK-1 cells infected with the facultative intracellular bacterium *Piscirickettsia salmonis*.

**Results:**

Effects of the DFO treatment and *P. salmonis* on SHK-1 cells were gaged by assessing cytopathic effects, bacterial load and activity, and gene expression profiles of eight immune biomarkers at 4- and 7-days post infection (dpi) in the control group, groups receiving single treatments (DFO or *P. salmonis*) and their combination. The chelator appears to be well-tolerated by host cells, while it had a negative impact on the number of bacterial cells and associated cytotoxicity. DFO alone had minor effects on gene expression of SHK-1 cells, including an early activation of *IL-1β* at 4 dpi. In contrast to few moderate changes induced by single treatments (either infection or chelator), most genes had highest upregulation in the infected groups receiving DFO. The mildest induction of *hepcidin-1* (antimicrobial peptide precursor and regulator of iron homeostasis) was observed in cells exposed to DFO alone, followed by *P. salmonis* infected cells while the addition of DFO to infected cells further increased the mRNA abundance of this gene. Transcripts encoding *TNF-α* (immune signaling) and *iNOS* (immune effector) showed sustained increase at both time points in this group while *cathelicidin-1* (immune effector) and *IL-8* (immune signaling) were upregulated at 7 dpi. The stimulation of protective gene responses seen in infected cultures supplemented with DFO coincided with the reduction of bacterial load and activity (judged by the expression of *P. salmonis* 16S rRNA), and damage to cultured host cells.

**Conclusion:**

The absence of immune gene activation under normal iron conditions suggests modulation of host responses by *P. salmonis*. The negative effect of iron deficiency on bacteria likely allowed host cells to respond in a more protective manner to the infection, further decreasing its progression. Presented findings encourage in vivo exploration of iron chelators as a promising strategy against piscirickettsiosis.

**Supplementary Information:**

The online version contains supplementary material available at 10.1186/s12917-021-02853-6.

## Background

Great number of proteins require iron as a cofactor to fulfil their diverse biological activities. Both pathogenic microorganisms and their vertebrate hosts use iron metalloproteins in electron transfer redox reactions associated with different energy metabolisms, DNA synthesis and other vital processes. In addition, iron is involved in the expression of virulence factors in bacteria, which must obtain it in order to establish an infection [1, 2]. The essentiality of iron makes it a target for competition at the host-pathogen interface, involving iron-mediated antimicrobial defenses and mechanisms that restrict availability of this essential nutrient to pathogens, as well as advanced iron acquisition strategies evolved by microorganisms as a counter measure [2]. Dysregulation and excess of iron not only promote microbial infections, but in addition, may be damaging to host cells because iron readily catalyzes production of free radicals through Fenton reaction. This consequently increases the risk of oxidative stress. Due to the need to tightly regulate iron, organisms deploy complex mechanisms for fine-tuning the acquisition of iron, maintenance of its homeostasis and metabolism during an infection [3].

As iron is required for virulence, proliferation and persistence of microorganisms, the deprivation of invading pathogens of iron by the host is often an effective antimicrobial mechanism [4–6]. This strategy is referred to as hypoferric, iron sequestration or iron withholding/withdrawal response and nutritional immunity, and is often observed in response to bacteria in mammals [7], and has been reported in fish [8, 9]. Hepcidin is the major iron-regulating hormone that is involved in the sequestration of iron from serum by macrophages, and is induced by proinflammatory cytokines, such as IL-1β and IL-6, activated by invading bacteria [10, 11]. It can also be modulated by other mediators of innate immunity like IL-8 and TNF-α [12, 13]. To make iron unavailable for microorganisms in circulation and other extracellular environments, the withdrawal response relies on iron binding by transferrin, haptoglobin and other proteins, and uptake of these iron-protein complexes by macrophages for safe intracellular storage (mainly within multimeric ferritin complexes). The resultant development of hypoferremia of inflammation may effectively starve invaders of the essential metal. However, if prolonged, anemia of inflammation may ensue, which is a condition characterized by the lack of iron required for incorporation into erythroid precursors despite the existence of normal iron stores [14]. In contrast, effective iron withholding responses against intracellular pathogens may involve suppression of the uptake of iron-protein complexes from circulation into macrophages and increase in the iron exportation from cells [15]. IFN-γ is the key cytokine that mediates efflux of intracellular iron levels [16]. It has been shown that antimicrobial responses that involve iron sequestration from macrophage residing pathogens protect the host against *Salmonella typhimurium*, *Mycobacterium tuberculosis* and *Chlamydia psittaci* [17–19]. However, the depletion of iron from macrophages may often also result in the development of anemia, which is in the long run negative for the host. Some intracellular pathogens, such as *Mycobacterium tuberculosis*, have evolved mechanisms for suppression of IFN-γ-regulated sequestration of iron [20], while *Salmonella* manipulates the host by activating hepcidin production which decreases iron export and maintains high level of intracellular iron, allowing these pathogens unimpeded utilization of iron stores inside the cells [17].

The iron withdrawal response is best documented in humans and mice, but appears to be an ancient antimicrobial mechanism predating the emergence of vertebrates [15]. Investigations of fish pathogens suggest that in response to the iron withholding strategy of the piscine host, complex mechanisms similar to those seen in pathogenic bacteria targeting mammalian hosts have also evolved. Under the iron limiting conditions, *Renibacterium salmoninarum*, the aetiological agent of bacterial kidney disease (BKD), activates its iron uptake machinery, which likely contributes to the higher toxicity against the salmon host cells [21]. Similar mechanisms of iron acquisition have also been described for *Tenacibaculum maritimum* [22]. Although viruses do not require iron to survive or proliferate, cells overloaded with iron may favor viral infections. Proliferation of the infectious pancreatic necrosis virus in the head kidney of Atlantic salmon resulted in gene expression changes suggestive of excessive intracellular iron load while associated increase in the production of free radicals could possibly be explained as a consequence of iron-mediated free radical production [23].

One of the most important diseases in the Chilean salmon aquaculture is piscirickettsiosis or Salmonid Rickettsial Septicemia (SRS), caused by the facultative intracellular bacterium *Piscirickettsia salmonis* [24]. As other similar pathogens, *P. salmonis* has developed strategies for manipulation of host protection mechanisms, including the expression of the ferric uptake regulator, which most likely controls the expression of virulence factors and iron acquisition machinery in this species, which is typical in a number of other bacteria [25]. Resistance of Atlantic salmon to SRS may at least partly be attributed to the regulation of iron witholding genes [9, 26]. Numerous pathogens have evolved iron acquisition strategies to overcome iron restriction imposed by the host. The synthesis and secretion of small iron chelators called siderophores, which have high affinity for iron bound by host proteins, is one such strategy [27, 28]. A recent paper by Calquin et al. [29] provided evidence that *P. salmonis* produces functional siderophores that are involved in the acquisition of iron. The complexity of interactions of *P. salmonis* with multiple host innate and adaptive responses is only beginning to be better understood [9, 30, 31]. Although a lot remains to be learned about the factors contributing to the development and establishment of SRS, based on the studies published so far, regulation of iron metabolism appears to have one of the key roles.

Here we evaluated the impact of artificially induced iron deprivation on the *P. salmonis* infection in Atlantic salmon SHK-1 cells mediated by the pharmacological treatment with the iron chelator deferoxamine mesylate (DFO).

## Results

### Characterization of cytopathic effect and bacterial load in infected SHK-1 cells

**Fig. 1:**
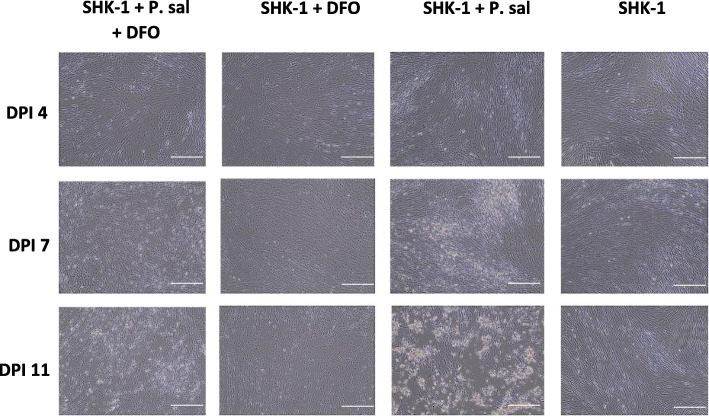
The evaluation of cytopathic effects in SHK-1 cells infected with *P. salmonis*. SHK-1 cells under four experimental conditions were evaluated microscopically for the cytopathic effects over 11 days, at 4, 7 and 11 days post infection (dpi). SHK-1 cells infected with *P. salmonis* were treated with DFO (SHK-1 + P. sal + DFO) and without it (SHK-1 + P. sal). Non-infected SHK-1 cells were treated with DFO (SHK-1 + DFO) to serve as a toxicity control. Positive (SHK-1 + P. sal) and negative controls (SHK-1) were used as references for treatment comparisons. For treatments with DFO, cells were pretreated with the chelator 24 h before infection. Images were taken with the EVOS® FL Color Imaging System (Invitrogen, Thermo Fisher Scientific Inc., Carlsbad, CA, USA) using 10X objective after 3 PBS-1X washes. The scale bar was added by using the image analysis ImageJ 1.37 software (National Institutes of Health, Bethesda, MD, USA)

To evaluate the *P. salmonis* infection under iron-limiting conditions, SHK-1 cells treated with or without DFO were microscopically monitored for 11 days (Fig. 1). The morphology of cells in the negative control (SHK-1) showed no apparent variation during the 11-day long evaluation period. In contrast, the cytopathic effect (CPE) was clearly visible at 7 days post infection (dpi) in the positive control (SHK-1 + P. sal), which became even more pronounced at 11 dpi. The timing of CPE in the present and similar studies [32, 33] may differ due to the selection of the EM-90-like strain and culture temperature and nutritional conditions. To test the DFO toxicity, non-infected SHK-1 cells exposed to DFO (SHK-1 + DFO) were monitored throughout the course of the study. Neither apparent damage in the cell monolayer nor high number of round and detached translucent cells were observed during the evaluation period. Further characterization of suspected minor morphological changes (more easily inspected in the Supplementary Figure 1 A-M, see images E and F) affecting the typical elongated shape of SHK-1 cells occurring at 7 and 11 dpi in response to DFO alone should be undertaken in future studies. The infected SHK-1 cells treated with DFO (SHK-1 + P. sal + DFO) showed less pronounced CPE compared to the positive control at 11 dpi.

**Fig. 2:**
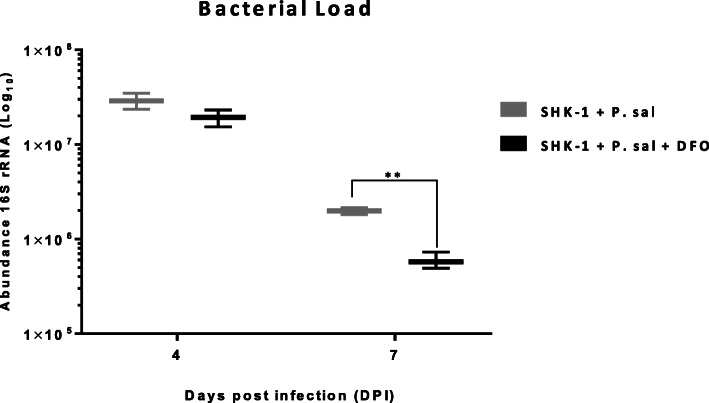
Bacterial load in infected SHK-1 cells. Bacterial load profiles of infected SHK-1 cells with (SHK-1 + P. sal + DFO) and without DFO (SHK-1 + P. sal) were evaluated by gene expression profiling of 16S rRNA gene of *P. salmonis* using RT-qPCR TaqMan**®** as described previously [1]. The profiles were shown as the abundance of rRNA at 4- and 7-days post infection (dpi) and effectively represent the result of bacterial number and transcriptional activity. Each time point was expressed as log_10_ and represents the mean ± SD from three independent experiments. The asterisks (**) indicate significant difference (*t*-test, *P* < 0.05)

The lower CPE of infected SHK-1 cells treated with DFO could be due to the decrease in the number of *P. salmonis* cells. Hence, profiling the expression of 16S rRNA gene as a proxy of the bacterial load was done to compare infected groups at 4 and 7 dpi (Fig. 2) (as it was determined in the pilot study, higher bacterial cell number results in a proportionally higher abundance of rRNA transcripts in a sample, see Method section). At 11 dpi, high cellular lyses observed in infected controls prevented a reliable measurement at this time point. While no difference was observed between infected SHK-1 cells with and without DFO at 4 dpi, a significant decrease in the expression of 16S rRNA was measured in infected cells under iron-limiting conditions (t-test, *p* < 0.05).

### Gene expression profiling

**Fig. 3:**
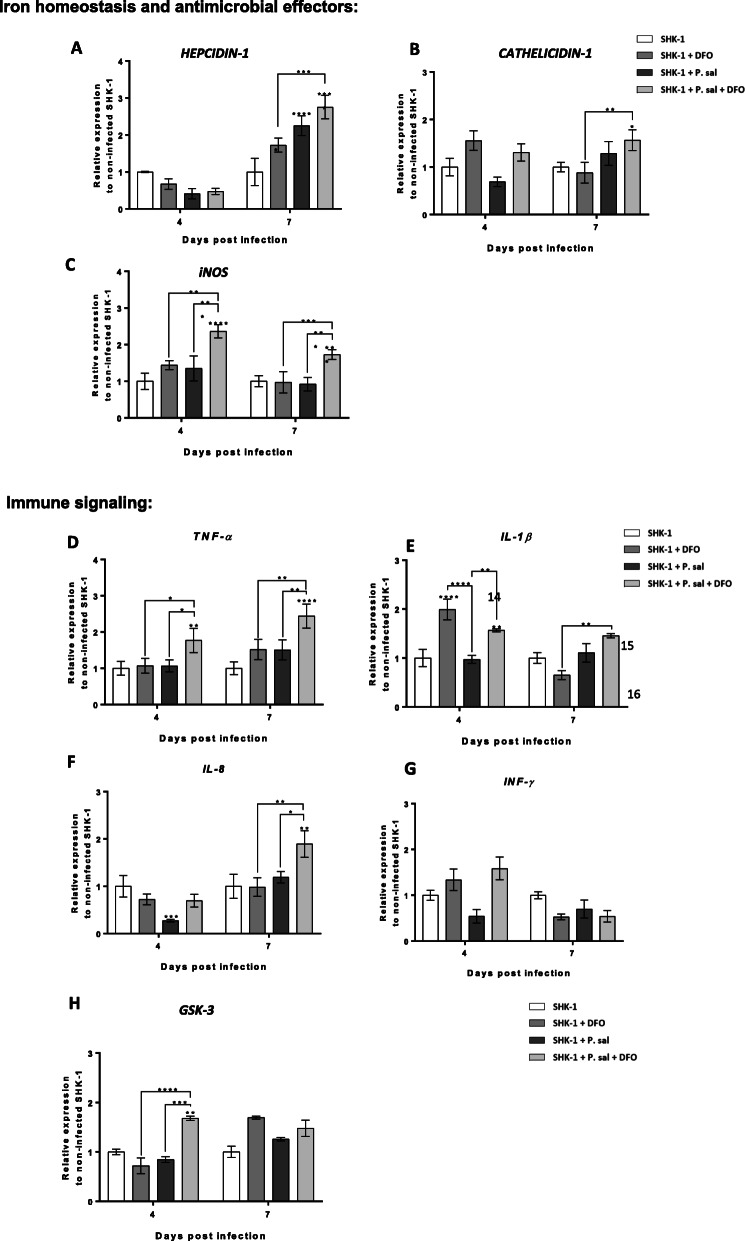
Profiles of genes involved in iron homeostasis, antimicrobial responses and immune signaling in SHK-1 infected with *P. salmonis* and exposed to DFO. Gene profiles were determined by using SYBR green RT-qPCR in SHK-1 cells under four conditions (SHK-1, SHK-1 + DFO, SHK-1 + P. sal and SHK-1 + P. sal + DFO) at 4- and 7-days post infection (dpi). Each test gene was normalized to *ELF-1α*. Relative expression is expressed as fold-change over the control condition (SHK-1, non-infected SHK-1 cells without DFO) for each time point. Data represent the mean ± SD of three independent experiments. The asterisks represent significant differences compared to control or between experimental conditions (indicated with n-line); *ρ < 0.05, **ρ < 0.01, ***ρ < 0.001, ****ρ < 0.0001

Responses of SHK-1 cells were evaluated by profiling the expression of eight genes encoding proteins involved in immune signaling (*TNF-α*, *IL-8*, *IL-1β*, *IFN-γ* and *GSK-3*0) and regulation of iron metabolism and antimicrobial effector responses (*hepcidin-1, cathelicidin-1* and *inducible nitric oxide synthase iNOS*) (Fig. 3). *Hepcidin-1*, which inhibits release of iron from intracellular macrophage pools and is also a precursor of an antimicrobial effector, responded in all test groups at 7 dpi. The lowest upregulation was seen in the infected cells with no additional treatment (SHK-1 + P. sal.) followed by slightly higher activation in the non-infected cells receiving the chelator (SHK-1 + DFO) while highest upregulation occurred when cells were exposed to both treatments (SHK-1 + P. sal + DFO) (Fig. 3a). A similar trend was observed for the antimicrobial peptide *cathelicidin-1* but the expression was significantly different only in infected cells treated with DFO (SHK-1 + P. sal + DFO) (Fig. 3b). Another antibacterial effector, *iNOS*, showed induction at both time points only in the infected cells exposed to the chelator (Fig. 3c). Profiling gene expression of four proinflammatory cytokines (*TNF-α*, *IL-8*, and *IL-1β*, *IFN-γ*) revealed significant upregulation of *TNF-α*, *IL-1β*, and *IL-8* (Fig. 3d, e and f, respectively) in the SHK-1 + P.sal + DFO group either at one or both time points while *IFN-γ* was not much affected by any treatment (Fig. 3g). The absence of *IFN-γ* response, which is one of the essential mediators of immune responses against intracellular pathogens [34] could be the result of the in vitro infection in SHK-1 cell line which is derived from leucocytes but is known to possess a number of macrophage properties [35].

## Discussion

The study evaluated the impact of iron chelator DFO applied as a preventive measure 24 h prior to exposure of SHK-1 cells to the EM-90-like *P. salmonis* strain. DFO could either act as a siderophore for *P. salmonis* and aggravate the infection course or limit iron access to bacterial cells and mediate protection. The findings were in favor of the partial DFO-mediated protection from infection as substantial reduction of CPE was observed in infected groups that received the chelator. Namely, infected cells that were exposed to DFO resulted in delayed and less pronounced damage of the cell monolayer. A strong iron-binding activity of DFO likely contributed to most of such clear-cut negative effect against *P. salmonis*. However, the chelator had a minor immunostimulatory effect in non-infected cells (*IL-1β* activation at 4 dpi), so it is possible that some antibacterial effect was exerted by early DFO-mediated upregulation of immune genes which were not tested in the present study. In support of this possibility, there is evidence showing that the antimalarial effect of DFO is due to the stimulation of effector immune mechanisms rather than to limitation of iron availability to the parasite [36]. However, its use as an adjunct antimalarial drug is linked to several adverse side effects [37]. DFO is widely used for the treatment of inherited disorders of iron overload with few toxic side effects [38–41], and it is considered an attractive therapeutant alone and in combination with other chelators for treatments against bacterial infections [42, 43]. Iron chelating properties of DFO have proven effective against *Porphyromonas gingivalis* by impairing bacterial growth and increasing the susceptibility of bacteria to other antimicrobial agents [44], and against *Pseudomonas aeruginosa* [45, 46]. The safety of administration of DFO to fish is yet to be addressed.

The DFO hydrophilic nature should make it difficult for the molecule to penetrate host cell membranes (except in hepatocytes) [6, 47], which could explain its negligible effect on gene expression in SHK-1 cells under our experimental conditions. The upregulation of *IL-1β* in non-infected salmon cells exposed to the chelator was likely an indication that cells detected and responded to lowered iron levels. The correlation between hepcidin and IL-1β expression has been previously reported in humans with iron deficiency [48]. Among its many functions, IL-1β contributes to the regulation of genes involved in the maintenance of iron homeostasis [49, 50]. The absence of IFN-γ activation, which promotes release of iron from cells to protect from intracellular pathogens, further supported the notion that the maintenance of intracellular iron pools under the conditions in the present study played a protective role and that increased entry of bacteria into cells likely did not occur. Changes in cellular morphology described in human leukemic cell lines, such as HL-60, were ascribed to antiproliferative and modulatory effects of DFO on cell differentiation [51–53]. DFO may have also caused a number of SHK-1 cells to change their elongated appearance to a monocyte-like form (Fig. 1), however, this remains to be studied in more detail in the future. Reports on HepG2 (human hepatoblastoma), HBG (human hepatocarcinoma) and HL-60 cell lines under DFO-mediated iron deprivation revealed its pro-apoptotic properties at concentrations above 100 μM [54, 55]. In theory, cell cycle may have been affected in non-infected SHK-1 cells in the present study. However, the hydrophilic nature of DFO, its short half-life [56], and low doses used likely precluded potential negative effects of DFO on salmon cells.

Endogenous protective responses of SHK-1 cells against the infection with *P. salmonis* were assessed by profiling genes involved in iron homeostasis, antimicrobial defenses and immune signaling. Upregulation of the master regulator of iron metabolism and antimicrobial peptide precursor *hepcidin-1* in all three treatment groups at 7 dpi indicated the need to increase intracellular iron stores and/or strengthen antimicrobial defenses. Its upregulation in the SHK-1 + DFO group might be a consequence of reduced extracellular iron levels caused by the chelator treatment, similar to the observed hepcidin induction that occurs under anemia in the teleost fish *Dicentrarchus labrax* [57]. It is not clear however if this strategy is protective in infected SHK-1 that did not receive DFO, as hepcidin mediates increase in the availability of iron to bacteria residing inside the cells. Data available so far suggest complex but overall positive effects of hepcidin activation in bacterial infections in fish. Induction of *hepcidin-1* by DFO in the liver, spleen and head kidney of Atlantic salmon undergoing *P. salmonis* infection coincided with the increase in survival as opposed to what occurred in groups supplemented with iron [58]. Hepcidin also exerted protective effect in grass carp against a disease caused by the extracellular *Flavobacterium* [59]. In contrast, the study of Atlantic salmon challenged by the facultative intracellular bacterium *Aeromonas salmonicida* did not find a protective effect of the iron withholding response. In fact, the suppression of hepcidin gene expression was found to be associated with an increase in survival [60].

Two other genes encoding antimicrobial effectors, *cathelicidin-1* and *iNOS*, as well as proinflammatory cytokines *TNF-α*, *IL-1β* and *IL-8* and *GSK-3* kinase involved in multiple cellular responses also showed highest level of expression in infected cells that were exposed to DFO and low level of activation by infection alone. The absence of upregulation of immune genes and early suppression of *IL-8* at 4 dpi likely reflect the capacity of *P. salmonis* to downplay at least a subset of proinflammatory cytokine responses, in line with the study of Alvarez et al. (2016) performed in the RTS11 trout cell line [30]. Of note, 4 dpi time point might not match the early induction of immune genes during the development of piscirickettsiosis in vivo [31, 61–63]. The observed antibacterial effect of DFO was likely further augmented by the pronounced increase in activation of immune biomarkers. TNF-α signaling mediates powerful antimicrobial responses against intracellular pathogens, including induction of apoptosis, killing of infected cells, and inhibition of pathogen replication though regulation of diverse host genes [64, 65] while IL-1β and IL-8 also induce a cascade of proinflammatory responses which aid in the clearance or containment of the pathogen [66].

**Fig. 4:**
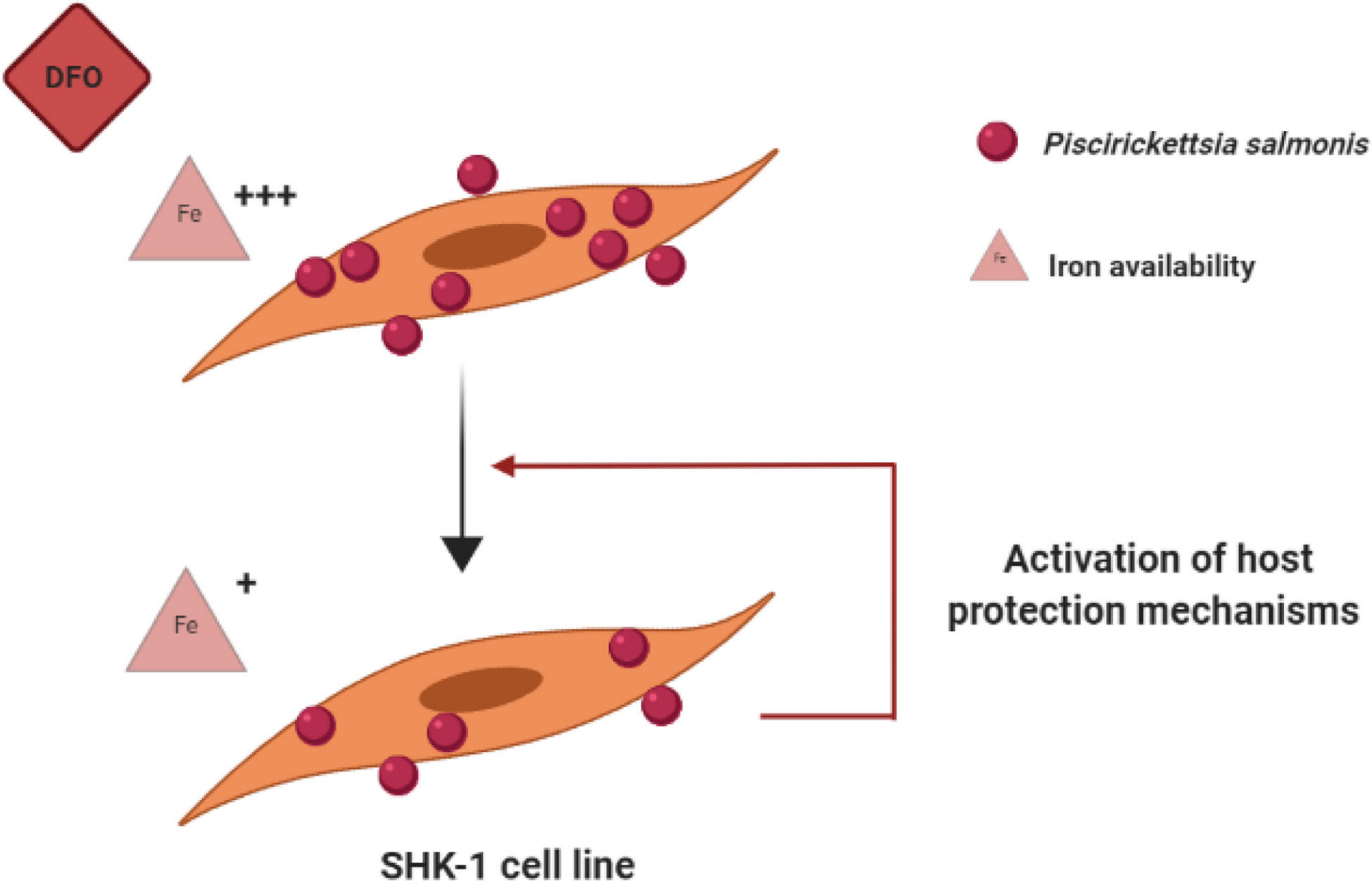
The summary of the effect of iron chelation on SHK-1 cells infected with *P. salmonis*. SHK-1 and *P. salmonis* cells grow using L-15 medium supplemented with 1xFBS as an iron source. Under these conditions, *P. salmonis* is able to activate virulence programs and effectively infect salmon cells. Limited access to iron by using iron chelator deferoxamine mesylate (DFO) impairs ability of bacteria to grow in the medium. The declining population of bacteria that managed the iron deprivation challenge must still face the host immune response. The DFO-mediated reduction in the number of *P. salmonis* cells allows for a stronger activation of protective host responses. This, in turn, further contributes to the clearance of bacterial cells

The study showed that exposing *P. salmonis* to the iron chelator DFO effectively reduces the number of bacterial cells over a period of 7 days and that the associated increase in activation of host protection mechanisms may further contribute to the antibacterial effect of the iron chelator. Figure 4 shows the likely sequence of events during the infection of SHK-1 cells by *P. salmonis*, which must survive nutrient deficient conditions and at the same time deal with the host immune response in order to establish an infection. Generated results encourage further in vivo exploration of iron chelators as a strategy against piscirickettsiosis.

## Conclusion

*P. salmonis* may be actively suppressing immune gene activation under normal iron conditions, as evidenced by the absence of activation of immune biomarkers used in this study and low expression profile of *IL-8* in infected cells not treated with the chelator. The decrease in the number of *P. salmonis* cells mediated by DFO likely allows host cells to respond to the infection in a more appropriate manner, further augmenting the negative effect of iron chelation on bacteria. The possible immunostimulatory effect of DFO on host cells warrants further investigation. Generated results encourage in vivo exploration of iron chelators as a promising strategy against piscirickettsiosis.

## Methods

### Piscirickettsia salmonis growth conditions

LF-89 and EM-90-like strains (kindly donated by VESO) of *P. salmonis* were grown aerobically on agar for 7–9 days at 22 °C. Bacteria in the cell-free medium were cultured on supplemented tryptic soy agar (TSA). This medium was prepared according to the following description: 22.5 g of TSA (Sigma-Aldrich, #22091), 2.5 g NaCl (Sigma-Aldrich, #S3014) and 2.5 g of glucose (Gibco, #15023–021) were mixed with 397.5 mL of distilled water and sterilized by autoclaving for 15 min at 121 °C. Once the autoclaved flask was cooled, the following ingredients were added: 25 mL of fetal bovine serum (FBS) (Gibco, #10437–028), 75 mL of 2% hemoglobine (DB, #211874) and 2.5 mL of 10% L-Cysteine (US Biologicals, #UB.C9005). Bacterial colonies were collected, transferred and resuspended into 10 mL of antibiotic-free Leibovitiz’s L-15 medium (Gibco, #11415056) and supplemented with 10% FBS (Gibco, #10270106). Bacteria were quantified by measuring the absorbance at a wavelength 600 nm using CO8000 cell density meter system (Biochrom US, #80300045).

### SHK-1 cell line and bacterial infection

The aim of the study was to establish a model of a relatively moderate infection which would be responsive to protective treatments, namely, an infection which could, at least partially, be rescued by iron chelation. SHK-1 cells with 55 passages (Sigma-Aldrich, #97111106) were grown in T-25 culture flasks (Thermo Fisher Scientific, #156367) with 10 mL of antibiotic-free Leibovitiz’s L-15 medium supplemented with 10% FBS at 18 °C until they reached ~ 90% confluence. In the pilot study, SHK-1 cells were infected with LF-89 and EM-90-like *P. salmonis* strains at MOI 25, 50, and 100, and monitored for the development of the CPE by microscopy (microscope EVO XL, Life Technologies) for 14 days. Noticeable CPE was observed with LF-89 at MOI 100 at 7 dpi, while CPE caused by EM-90-like strain at MOI 100 was already visible at 4 dpi. The comparison of the two strains resulted in the selection of EM-90-like as a more suitable strain for the purpose of studying the effect of iron chelation under the experimental conditions in this study. Cells were infected with the EM-90-like strain of *P. salmonis* at MOI 100 when cells reached 90% confluence. The development of CPE using a microscope (Invitrogen, #AMEFC4300) was recorded at 4, 7 and 11 dpi.

### Study of iron-limited condition in infected SHK-1 cells

To study the deficiency of iron, iron-limited conditions were generated by administration of deferoxamine mesylate salt (DFO, Sigma-Aldrich, #D9533). DFO was dissolved in sterile water (stock solution), passed through a 0.22 μm sterile filter (Biofilter, #FMC201030) and added to the T-25 culture flasks until final DFO concentration of 100 μM 24 h prior to the infection with *P. salmonis* was achieved. The experimental and control conditions were the following: SHK-1 + DFO (non-infected cells exposed to 100 μM DFO) and SHK-1 + P. sal + DFO (SHK-1 cells infected with *P. salmonis* and treated with 100 μM DFO). Positive control was SHK-1 + P. sal (infected SHK-1 cells with *P. salmonis* but without DFO). Negative control was SHK-1 (non-infected SHK-1 cells without DFO).

### RNA extraction

The total RNA extraction from SHK-1 cells was performed by using TRIzol method (Invitrogen, #15596026) and PureLink RNA Mini Kit (Invitrogen, #12183025). L-15 Leibovitz (Invitrogen. Catalog #1145114) media was removed and cells were washed three times with 1 mL of sterile 1X PBS (Sigma-Aldrich, #P4244). Cells were then lysed by adding 1 mL TRIzol reagent while making sure that the reagent coats the entire surface of the flask. After 15 min of incubation at room temperature, the lysate was scrapped, resuspended with the pipette and then transferred into 1.5- mL RNAase-free Eppendorf tubes with 0.2 mL of chloroform following the PureLink protocol. The mixture was passed through the PureLink columns and total RNA was extracted according to manufacturer’s instructions. RNA was quantified by spectrophotometry (Thermo Fisher Scientific, #NanoDrop-ONE-W), and RNA integrity was determined by electrophoresis on 1% agarose gel stained with GelRed (Biotium, #31010).

### Bacterial load quantification

In addition to CPE determined by microscopy which can be considered a somewhat subjective method, differences between study groups related to the severity and progression of infection were evaluated by the quantification of the relative gene expression of 16S rRNA gene by qRT-PCR using TaqMan™ probe with AgPath-ID™ One-Step RT-PCR Kit (Applied biosystems, Life Technologies, Waltham, Ma, USA). The suitability of using RNA versus DNA extract as a substrate for the quantification of the progression of infection was evaluated in a pilot study (data not shown). As expected, higher abundance of rRNA was measured when RNA was used as a substrate in comparison to the DNA substrate, clearly revealing contribution of transcription to the result. The generated values thus represent a cumulative result of the *P. salmonis* cellular proliferation and transcriptional activity. However, since Ct values generated by using RNA and DNA substrates were highly correlated, results produced when using RNA as a substrate can be considered a very good proxy of the bacterial load. Using RNA saves resources as new cultures would have to be grown solely for the purpose of DNA extraction, which could in addition introduce culture batch to batch variability. Noteworthy, proliferation and transcriptional activity of rRNA genes may not highly correlate under experimental conditions different from the ones in the present study, so suitability of using RNA as a substrate to infer progression of infection should always be determined in a pilot study. The PCR reaction was performed in a final volume of 20 μL using 100 ng of total RNA under conditions as follows: 10 min at 50 °C for reverse transcription (RT), followed by 10 min at 95 °C for RT inactivation and initial denaturation and 45 cycles of 15 s at 95 °C and 45 s at 60 °C for amplification. The relative abundance of 16S rRNA gene was calculated and expressed as log_10_, as previously described [67].

### Gene expression and statistical analysis

The relative gene expression of eight biomarkers mostly involved in innate immune responses was performed using SensiMix™ SYBR™ and Fluorescein kit (Bioline, Taunton, MA) according to manufacturer’s instructions. The synthesis of first strand cDNA was performed by reverse transcription using AffinityScript QPCR cDNA Synthesis Kit (Agilent Technologies, Santa Clara, CA, USA). Samples were diluted to 100 ng and used as template for RT-qPCR analysis. Reactions were performed in 7500 Fast Real-Time PCR System (Applied Biosystems, Life Technologies, Waltham, MA, USA) and PCR program was as follows: 95 °C for 10 min, followed by 40 cycles at 90 °C for 10 min, 60 °C for 30 s and 72 °C for 15 s. Primer sequences are shown in Table 1. Melting curves of the amplicons were analyzed to confirm unique PCR product. The relative expressions were calculated using comparative Ct method following the protocol established by [70]. Data were normalized to elongation factor 1-alpha (*ELF-1α*) and relative to the negative control (non-infected SHK-1 cells not exposed to DFO) for each time point. GraphPrism 7 (GraphPad Software, La Jolla, CA, USA) was used for the statistical analysis of the databy using a *t*-test (bacterial load quantification) and ANOVA (gene expression) followed by post-hoc Tukey test to identify differences between groups.

**Table 1:** Primers used for gene expression analysis by RT-qPCR

Gene name or symbol	Accession	Function related	Primers (5′- > 3′)	Reference
*HEPCIDIN-1*	BT125319	Iron regulator, Antimicrobial	F:ATGAATCTGCCGATGCATTTC	This study
			R: AATGGCTTTAGTGCTGGCAG	
*CATHELICIDIN-1 (CATH)*	AY360357	Antimicrobial	F: AGACTGGCAACACCCTCAAC	[68, 69]
			R: TTGCCTCTTCTTGTCCGAAT	
*iNOS*	AF088999	Antimicrobial	F: GGAGAGCCTTCTGGTTG	[69]
			R: ACCTTAACTTGTTCCTGAGATAC	
*TNF-α*	NM_001123589	Immune signalling	F: AGGTTGGCTATGGAGGCTGT	[63]
			R: TCTGCTTCAATGTATGGTGGG	
*IL-1β*	NM_001123582	Immune signalling	F: ATCACCATGCGTCACATTGC	[63]
			R: GTCCTTGAACTCGGTTCCCA	
*IL-8*	NM_001140710	Immune signalling	F: GGCCCTCCTGACCATTACT	[63]
			R: ATGAGTCTACCAATTCGTCTGC	
*IFN-γ*	AY795563	Immune signalling	F: CTAAAGAAGGACAACCGCAG	[63]
			R: CACCGTTAGAGGGAGAAATG	
*GSK-3*	BT049486.1	Immune signalling	F: AAAAGAAGTGGACGCGTTGG	This study
			R: GTTACTACTGCTGCAGTTGCTG	
*ELF-1α*	AF321836	Normalizer	F: CTGGCACTTTCACTGCTCAAG	This study
			R: CAACAATAGCAGCGTCTCCA	

## Supplementary Information

**Additional file 1: Supplementary file 1 A-M.** The evaluation of cytopathic effects in SHK-1 cells infected with *P. salmonis*. Separate images of cell cultures under four experimental conditions at days 4, 7 and 11 (dpi): non-infected SHK-1 not treated with DFO (SHK-1), non-infected SHK-1 cells treated with DFO (SHK-1 + DFO), infected SHK-1 cells not treated with DFO (SHK-1 + P. sal) and SHK-1 cells infected with *P. salmonis* and treated with DFO (SHK-1 + P. sal + DFO). Images were taken with the EVOS® FL Color Imaging System (Invitrogen, Thermo Fisher Scientific Inc., Carlsbad, CA, USA) using 10X objective after 3 PBS-1X washes. The scale bar was added by using the image analysis ImageJ 1.37 software (National Institutes of Health, Bethesda, MD, USA). Scale bar = 260 μM.

## Data Availability

The datasets used and/or analyzed during the current study are available from the corresponding author on reasonable request.

## Abbreviations

BKD: Bacterial Kidney Disease
SRS: Salmon Rickettsial Septicemia
DFO: Deferoxamine mesylate
SHK-1: Salmon Head Kidney-1
CPE: Cytopathic effect
dpi: days post infection
CATH: Cathelicidin-1
iNOS: inducible nitric oxidase
TNF-α: Tumor necrosis factor alpha
IL-1β: Interleukin 1 beta
IL-8: Interleukin 8
IFN-γ: Interferon gamma
GSK-3: Glycogen synthase kinase-3 binding protein-like
ELF-1α: Elongation factor 1-alpha
