# Supplementary material for: “Limiting access to iron decreases infection of Atlantic salmon SHK-1 cells with bacterium Piscirickettsia salmonis”
Source: BMC Vet Res. 2021 Apr 13;17:155. doi: 10.1186/s12917-021-02853-6 (PMC8043062; doi:10.1186/s12917-021-02853-6)
Supplement: Supplementary file 1 — Additional file 1: Supplementary file 1 A-M. The evaluation of cytopathic effects in SHK-1 cells infected with P. salmonis. Separate images of cell cultures under four experimental conditions at days 4, 7 and 11 (dpi): non-infected SHK-1 not treated with DFO (SHK-1), non-infected SHK-1 cells treated with DFO (SHK-1 + DFO), infected SHK-1 cells not treated with DFO (SHK-1 + P. sal) and SHK-1 cells infected with P. salmonis and treated with DFO (SHK-1 + P. sal + DFO). Images were taken with the EVOS® FL Color Imaging System (Invitrogen, Thermo Fisher Scientific Inc., Carlsbad, CA, USA) using 10X objective after 3 PBS-1X washes. The scale bar was added by using the image analysis ImageJ 1.37 software (National Institutes of Health, Bethesda, MD, USA). Scale bar = 260 μM. [file 12917_2021_2853_MOESM1_ESM.pptx]

## Slide 1
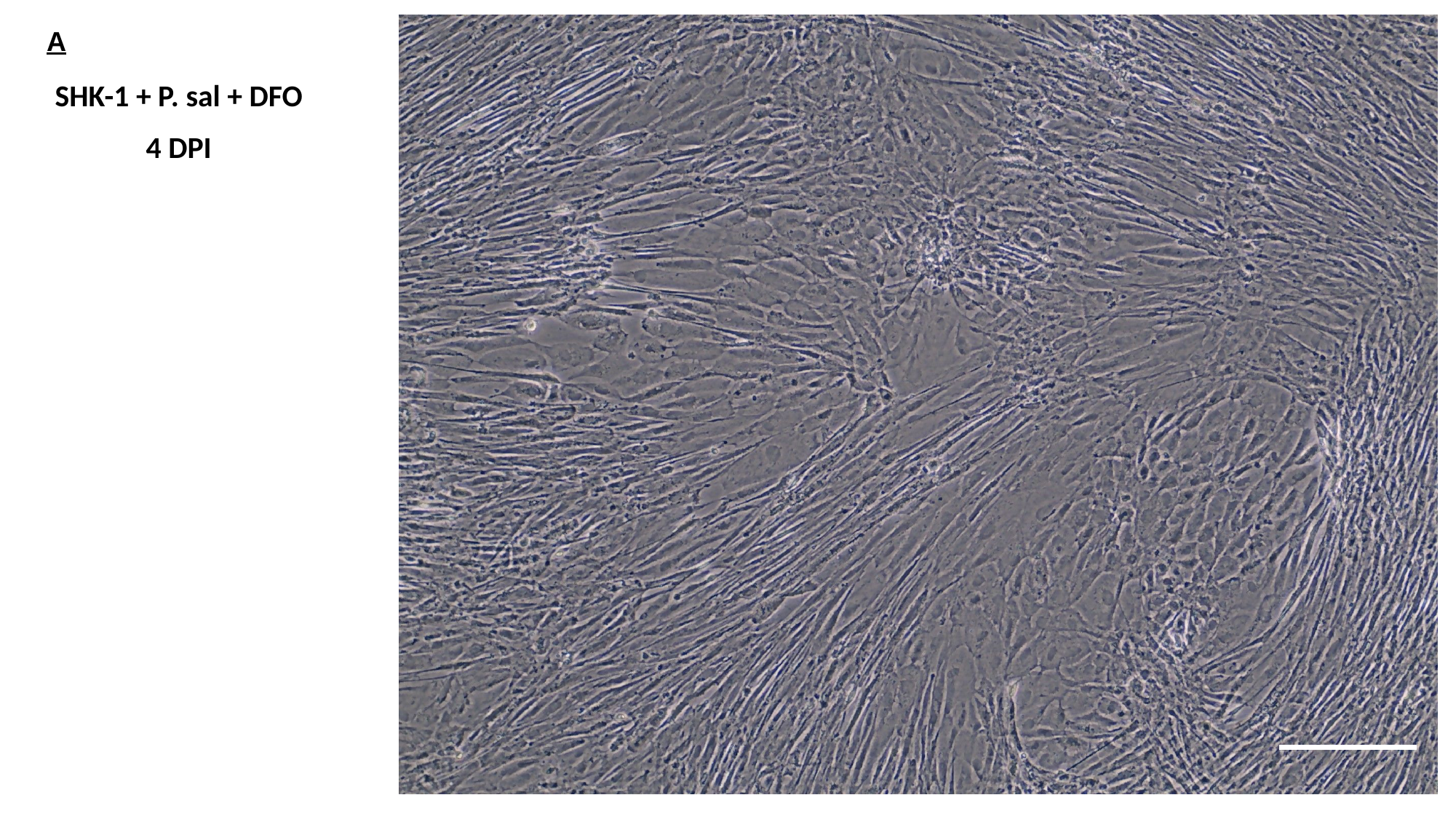

A
SHK-1 + P. sal + DFO
4 DPI

## Slide 2
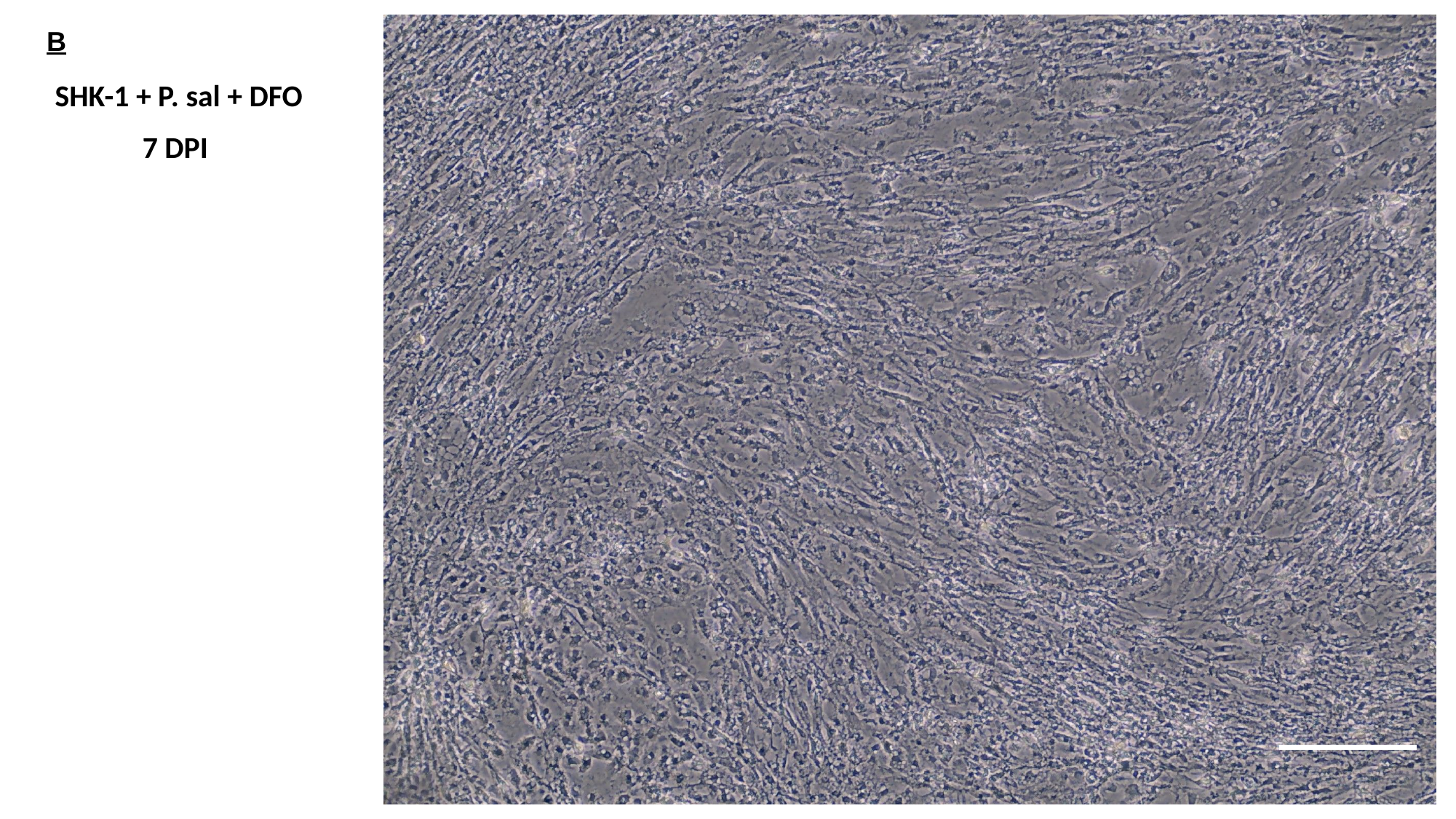

B
SHK-1 + P. sal + DFO
7 DPI

## Slide 3
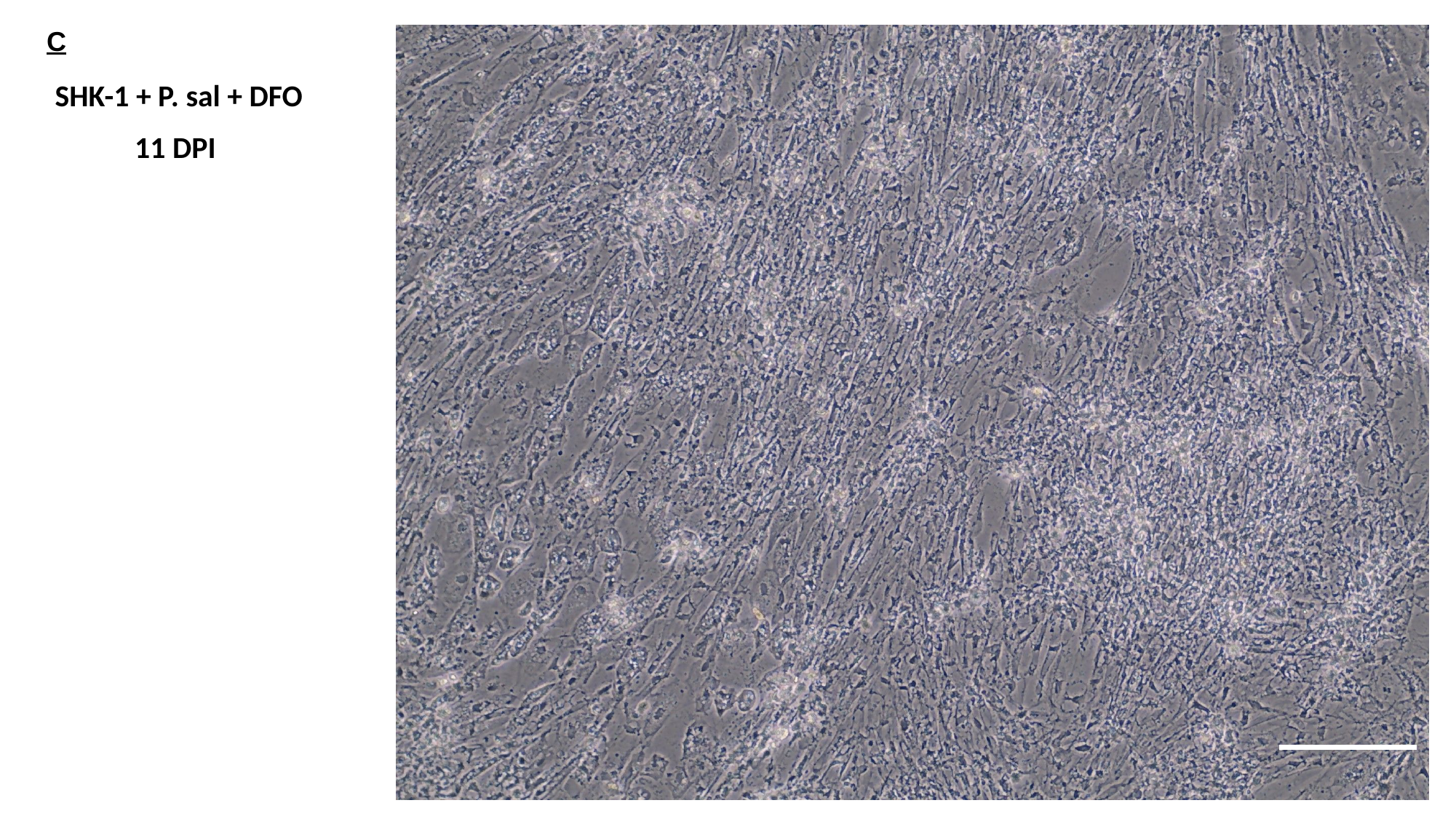

C
SHK-1 + P. sal + DFO
11 DPI

## Slide 4
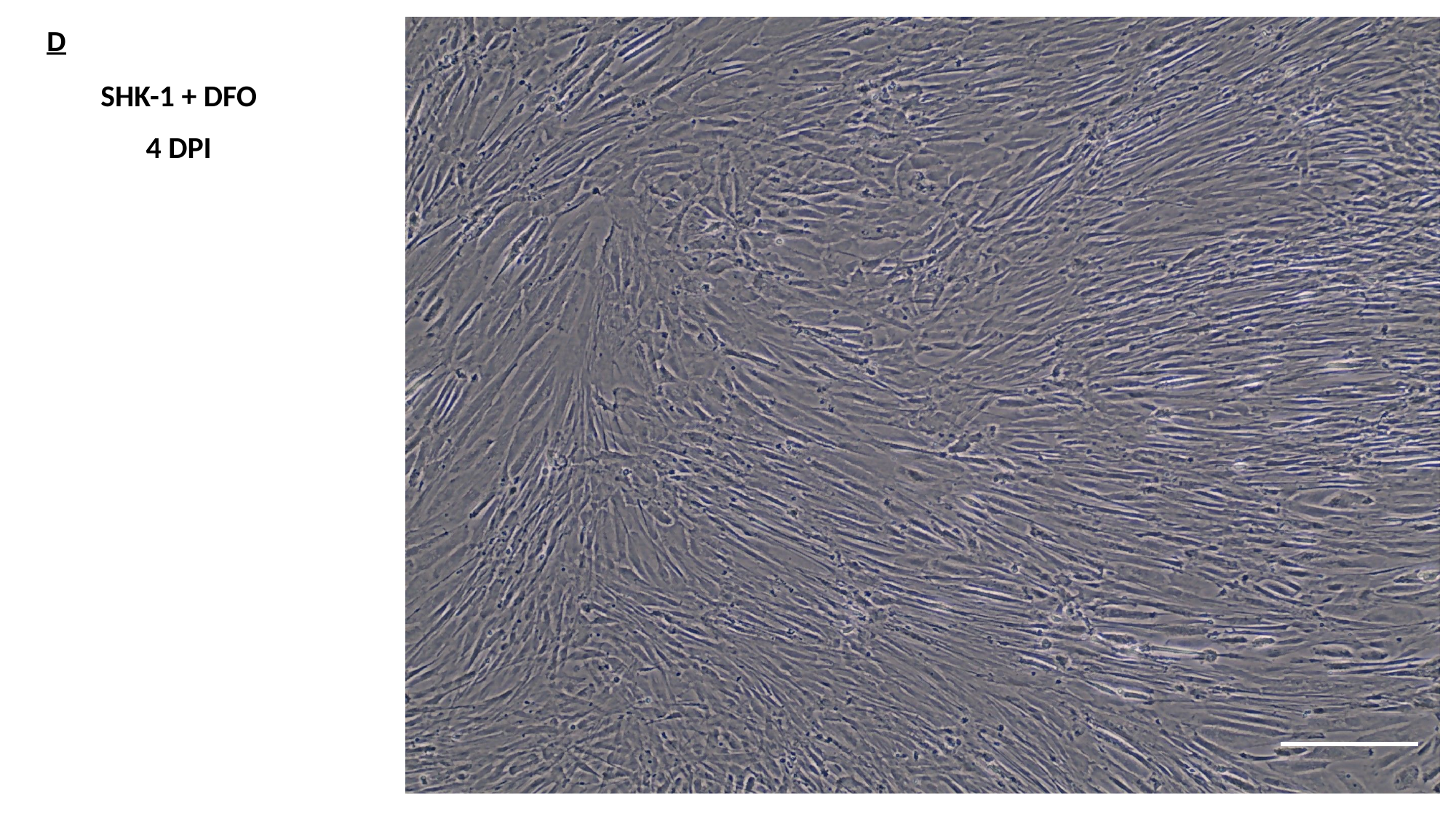

D
SHK-1 + DFO
4 DPI

## Slide 5
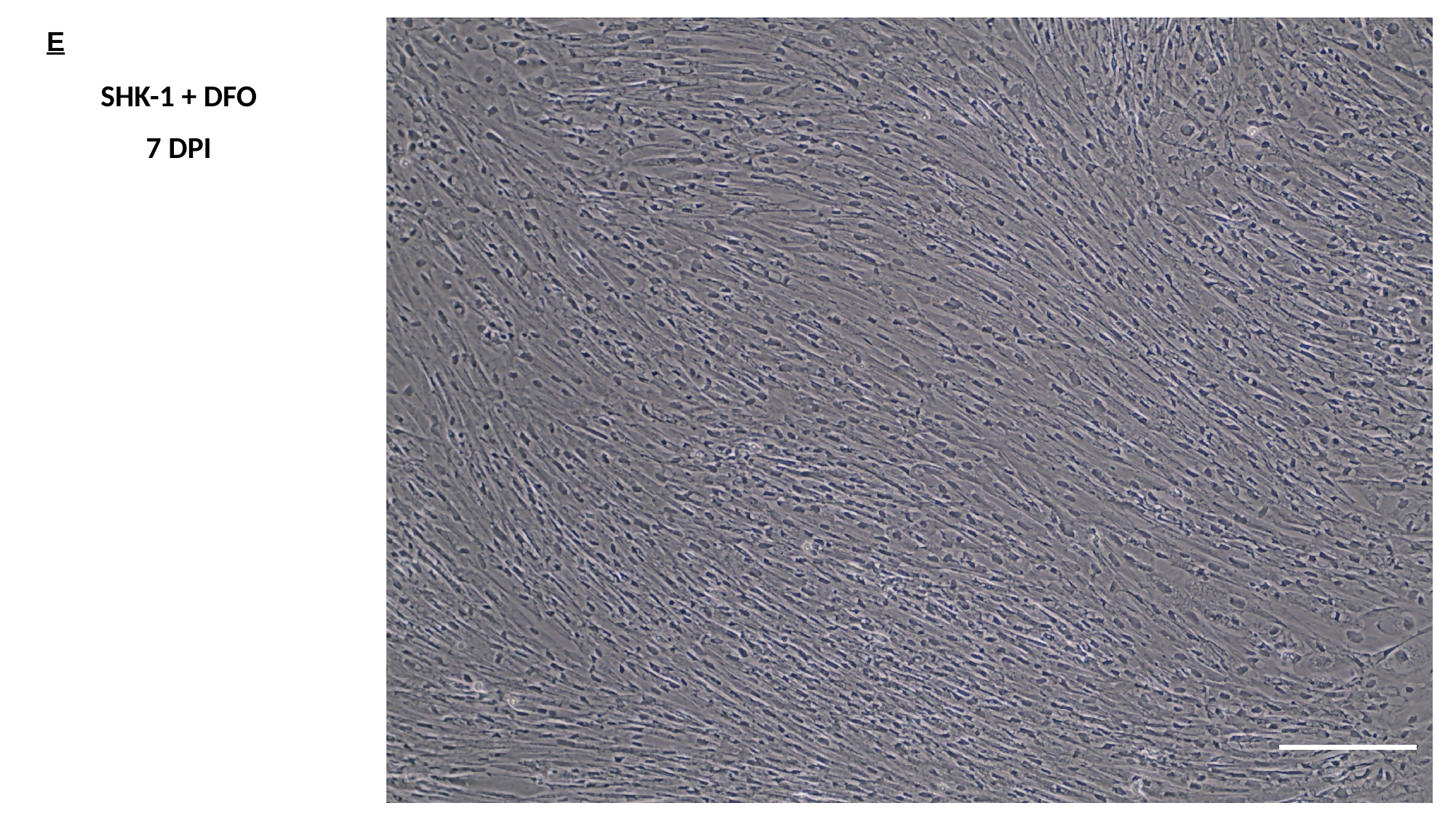

E
SHK-1 + DFO
7 DPI

## Slide 6
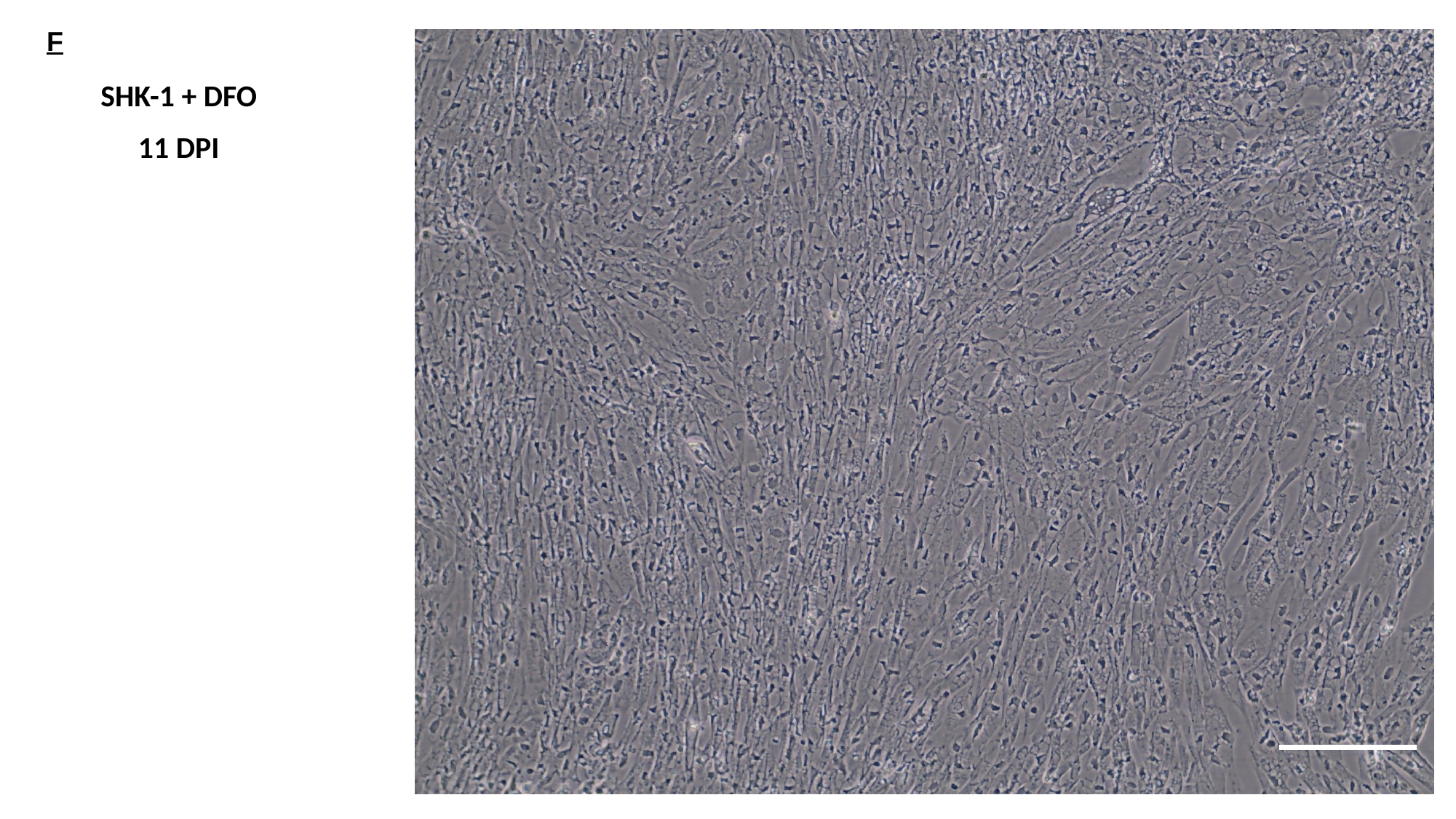

F
SHK-1 + DFO
11 DPI

## Slide 7
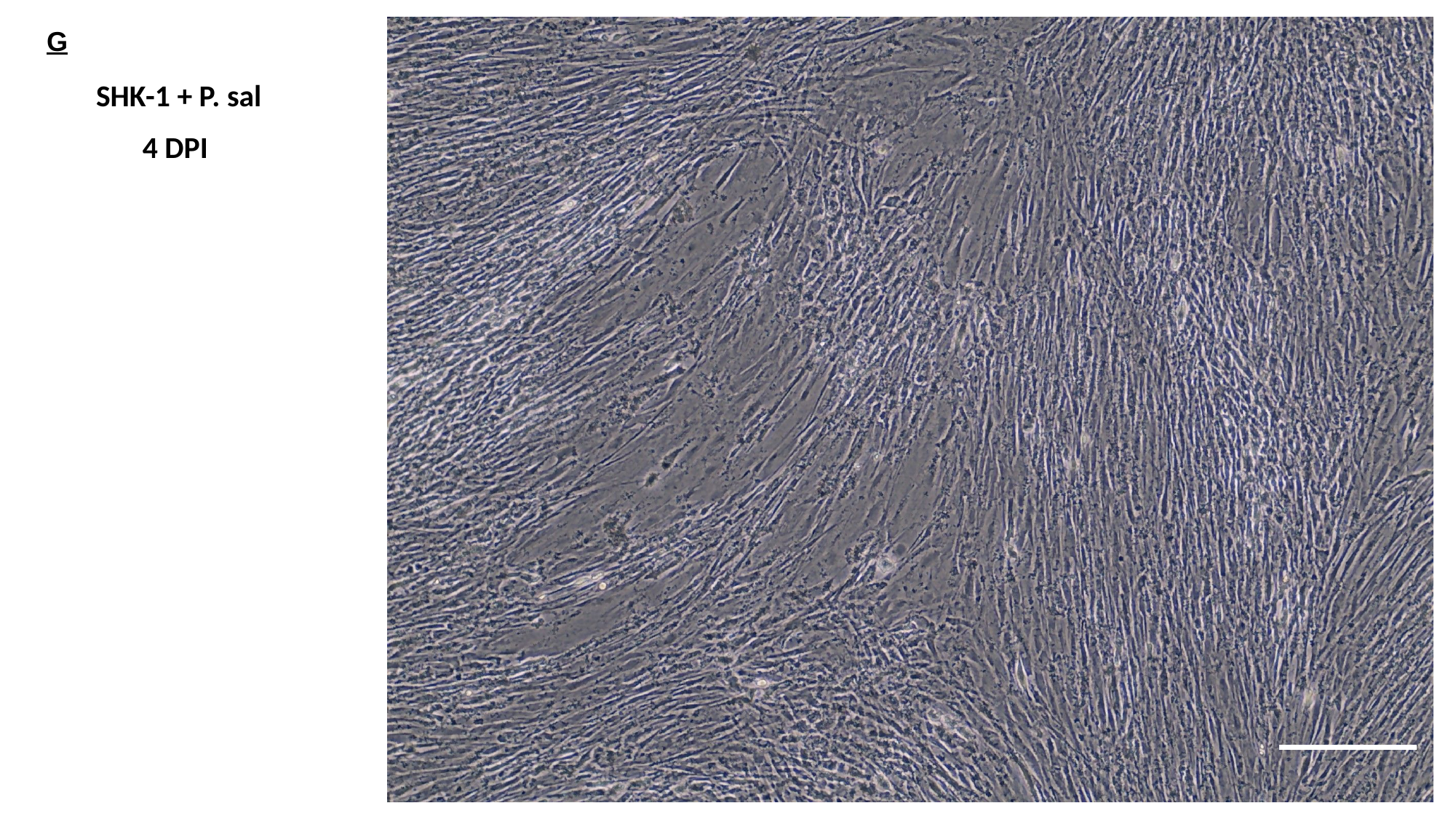

G
SHK-1 + P. sal
4 DPI

## Slide 8
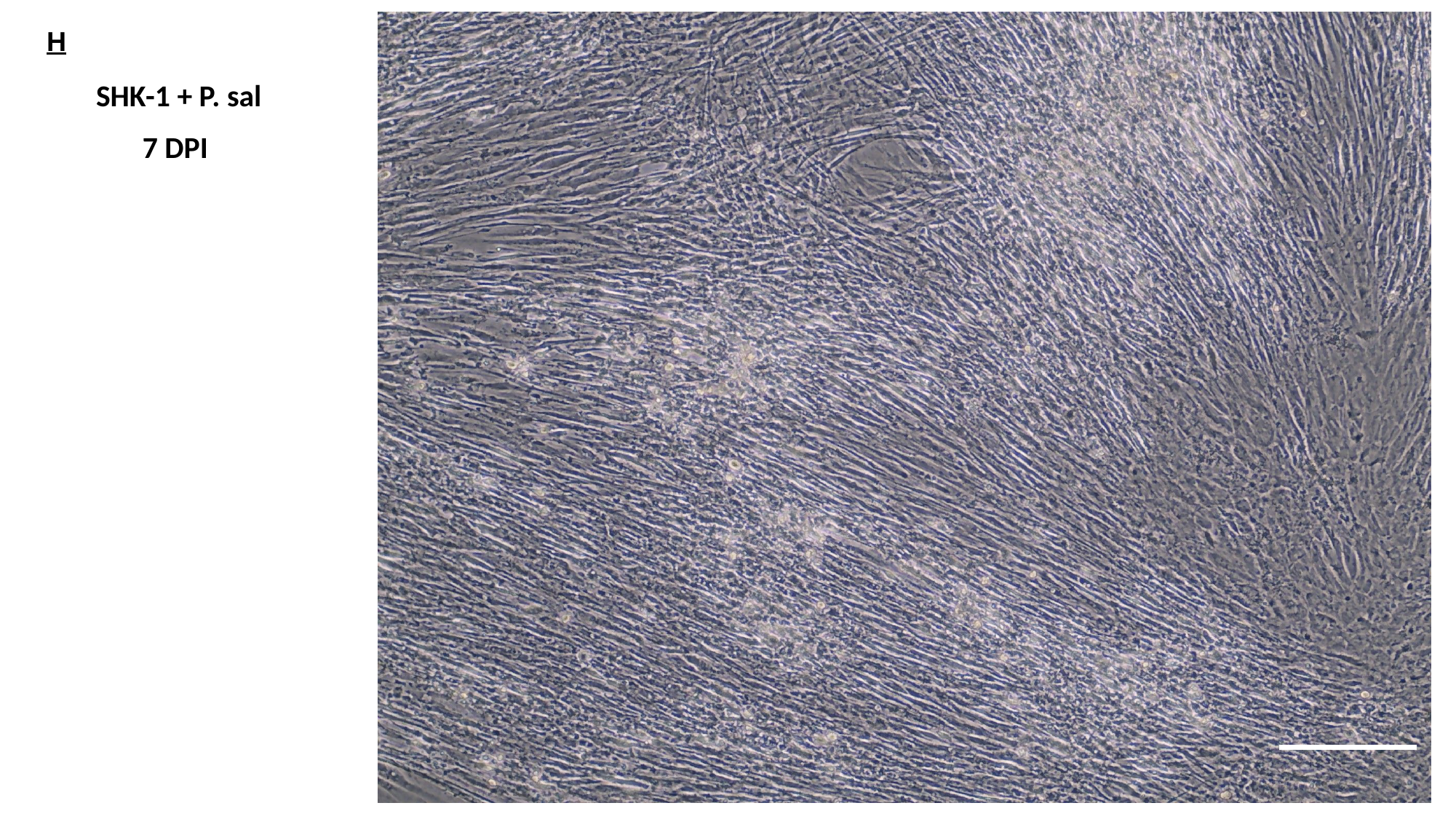

H
SHK-1 + P. sal
7 DPI

## Slide 9
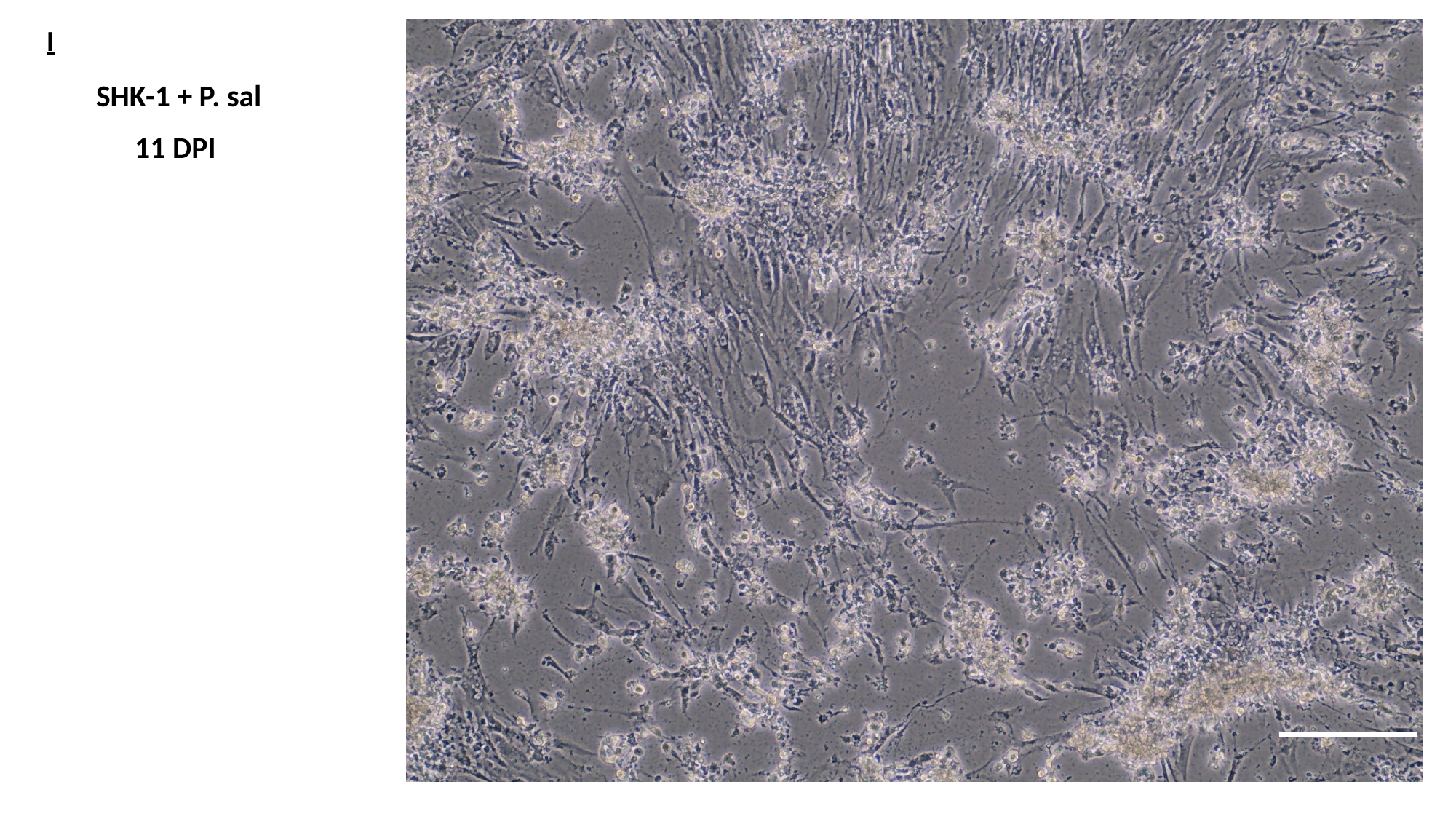

I
SHK-1 + P. sal
11 DPI

## Slide 10
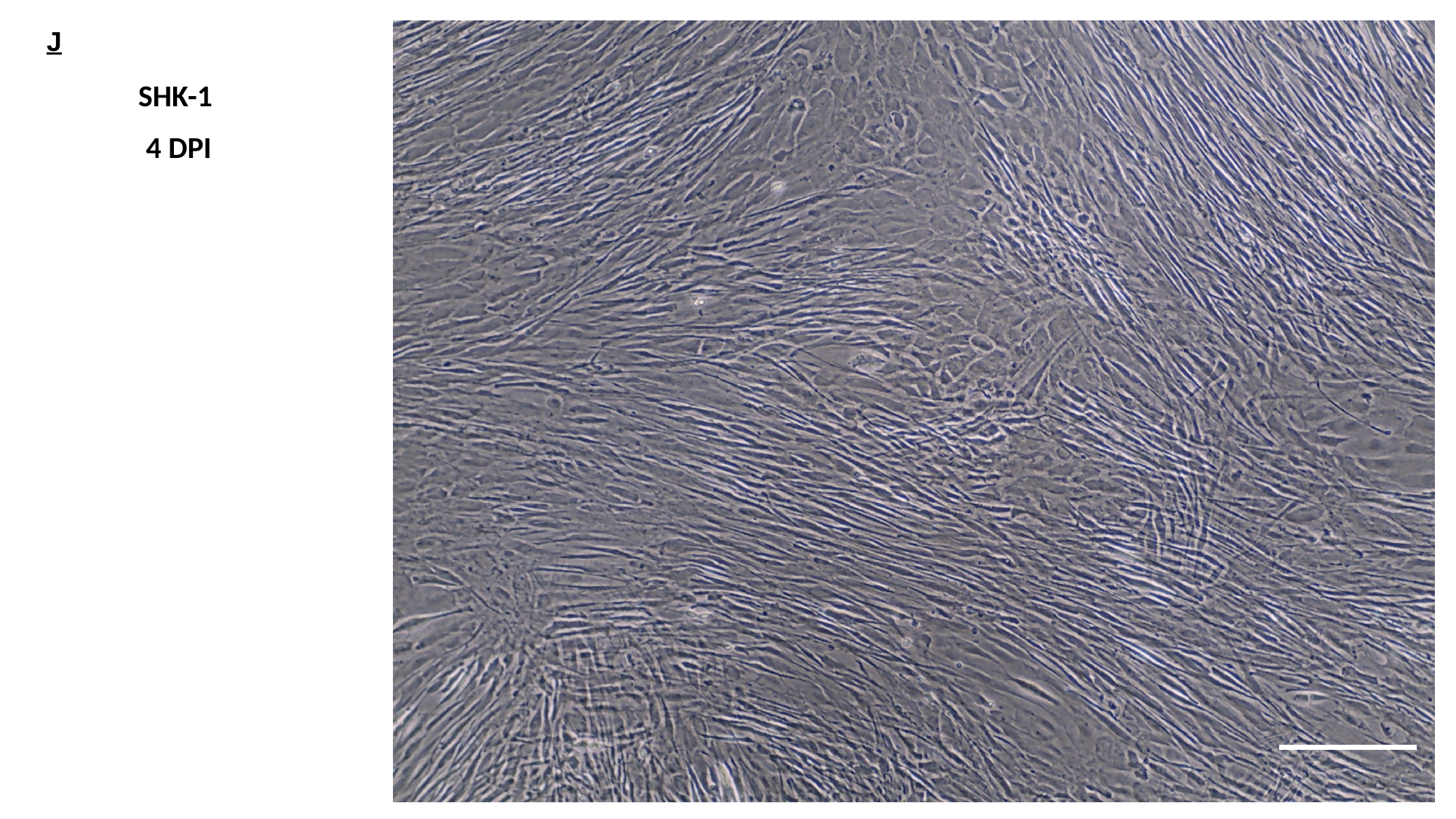

J
SHK-1
4 DPI

## Slide 11
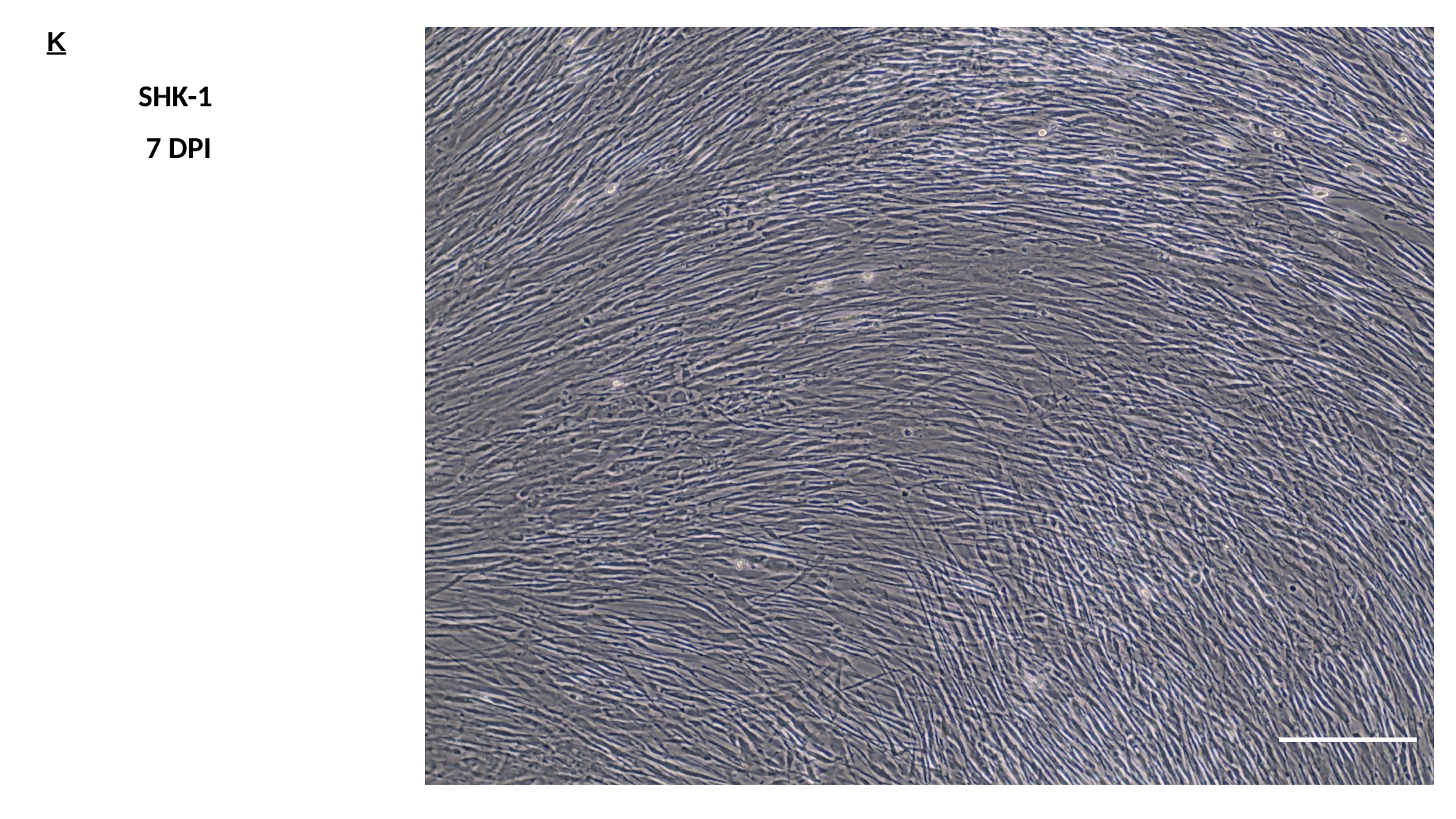

K
SHK-1
7 DPI

## Slide 12
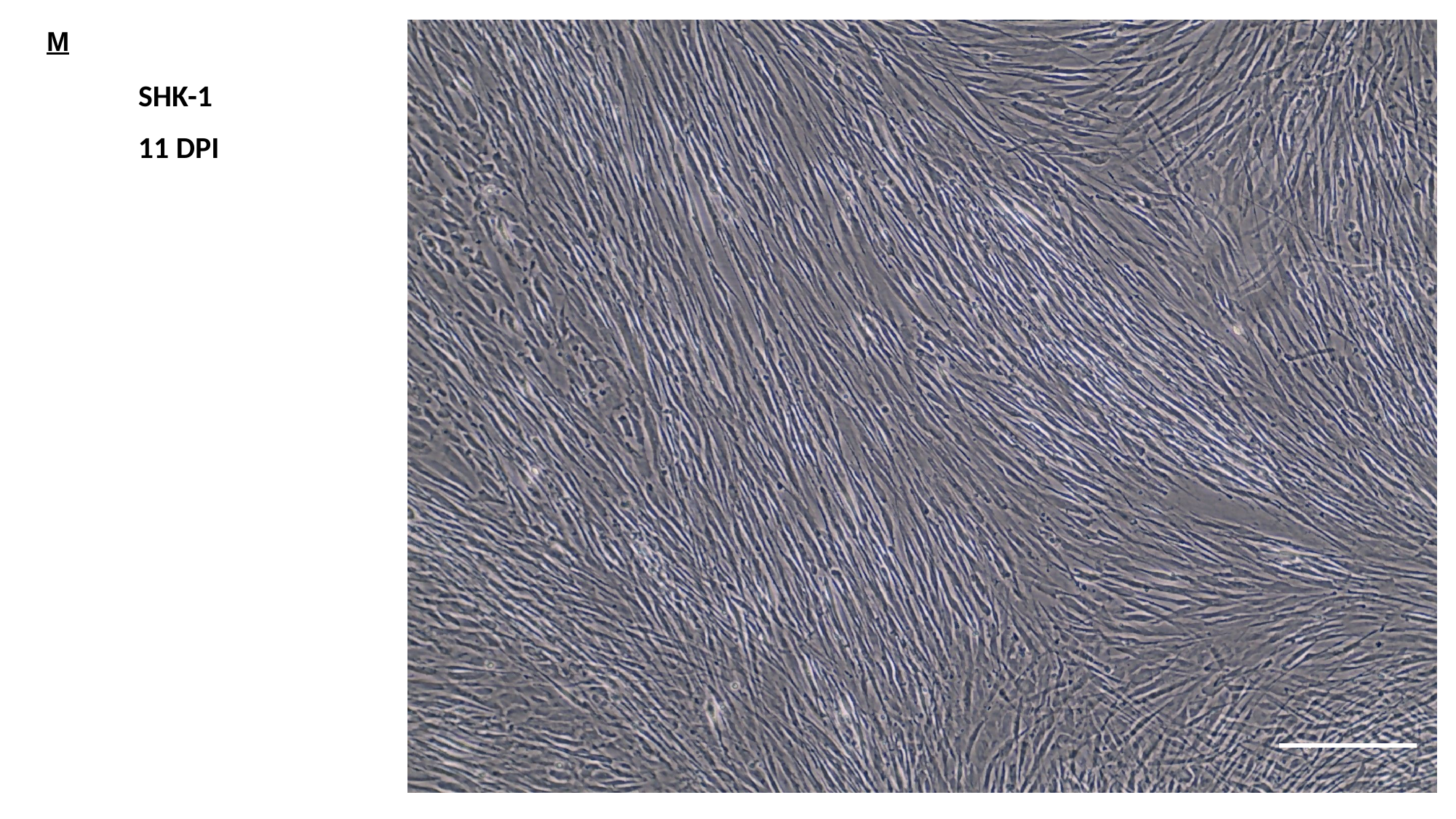

M
SHK-1
11 DPI

## Slide 13
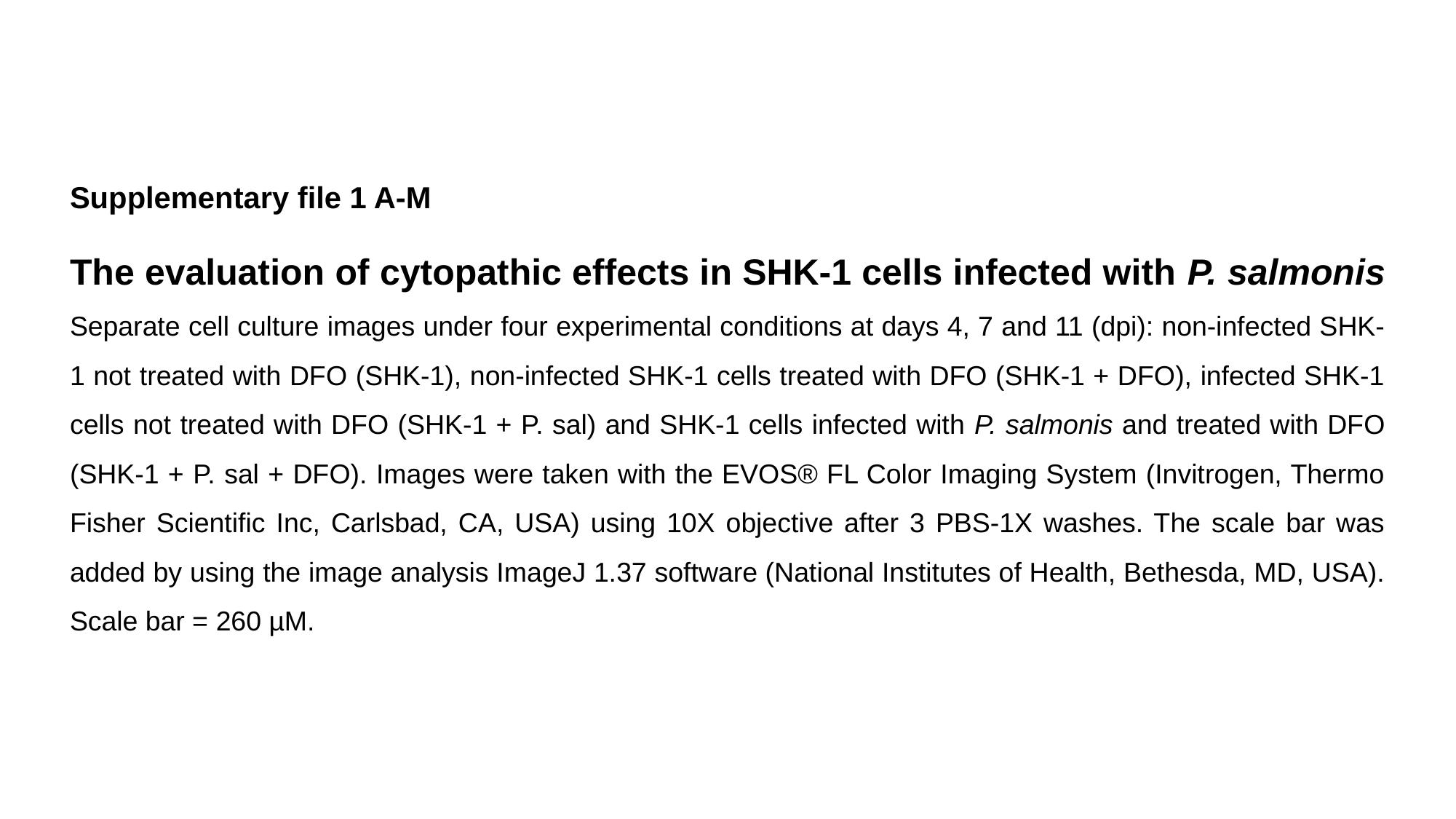

Supplementary file 1 A-M
The evaluation of cytopathic effects in SHK-1 cells infected with P. salmonis
Separate cell culture images under four experimental conditions at days 4, 7 and 11 (dpi): non-infected SHK-1 not treated with DFO (SHK-1), non-infected SHK-1 cells treated with DFO (SHK-1 + DFO), infected SHK-1 cells not treated with DFO (SHK-1 + P. sal) and SHK-1 cells infected with P. salmonis and treated with DFO (SHK-1 + P. sal + DFO). Images were taken with the EVOS® FL Color Imaging System (Invitrogen, Thermo Fisher Scientific Inc, Carlsbad, CA, USA) using 10X objective after 3 PBS-1X washes. The scale bar was added by using the image analysis ImageJ 1.37 software (National Institutes of Health, Bethesda, MD, USA). Scale bar = 260 µM.
